# Health System Factors Affecting the Experience of Non-Invasive Ventilation Provision of People with Neuromuscular Disorders in New Zealand

**DOI:** 10.3390/ijerph20064758

**Published:** 2023-03-08

**Authors:** Meredith A. Perry, Bernadette Jones, Matthew Jenkins, Hemakumar Devan, Alister Neill, Tristram Ingham

**Affiliations:** 1 Centre for Health, Activity and Rehabilitation Research (CHARR), School of Physiotherapy, Dunedin 9016, New Zealand; 2 Department of Medicine, University of Otago—Wellington, Wellington 6242, New Zealand; 3 Foundation for Equity & Research New Zealand, Wellington 6147, New Zealand; 4 Department of Respiratory Medicine, Te Whatu Ora Capital, Coast and Hutt Valley, Wellington 6140, New Zealand

**Keywords:** qualitative, neuromuscular disorders, non-invasive ventilation, equity, health systems, healthcare quality variation

## Abstract

Non-invasive ventilation (NIV) is a critical therapy for many patients with neuromuscular disorders (NMD), supporting those with respiratory failure to achieve adequate respiration and improve their quality of life. The aim of this study was to explore the experiences of access to, consent, uptake, maintenance and safe use of non-invasive ventilation by people with NMD. Semi-structured individual interviews were conducted with 11 people with NMD, each using NIV for more than 12 months. A critical realism ontological paradigm with contextualism epistemology guided the Reflexive Thematic Analysis. An Equity of Health Care Framework underpinned the analysis. Three themes were interpreted: Uptake and informed consent for NIV therapy; Practicalities of NIV; and Patient-clinician relationships. We identified issues at the system, organization and health professional levels. Conclusions: We recommend the development of national service specifications with clear standards and dedicated funding for patients with NMD and call on the New Zealand Ministry of Health to proactively investigate and monitor the variations in service delivery identified. The specific areas of concern for patients with NMD suggest the need for NMD-related NIV research and service provision responsive to the distinct needs of this population.

## 1. Introduction

A major concern for people living with neuromuscular disorders (NMD) is the associated respiratory muscle weakness and chronic respiratory failure, which occur over time [1,2]. Respiratory failure is often first noticeable during sleep, when the drive to breathe is lower and the upper airway more collapsible. This can elicit symptoms of fatigue, headaches and day somnolence [3]. With disease progression, respiratory failure may also occur during the daytime, severely restricting activities of daily living and resulting in repeated hospital admissions for respiratory infections [3,4]. Such failure can cause serious health consequences and represents a major source of morbidity and mortality in people with NMD [4,5]

Non-invasive ventilation (NIV) is the gold standard treatment for chronic respiratory failure that can be delivered during sleep (via nose or mouth and nose interface) and in the daytime (via mouthpiece or nose interface) [6]. Research has shown that NIV reduces the need for hospitalization and increases survival among people living with some neuromuscular disorders [7,8,9,10,11]. In addition, research has demonstrated NIV use improves sleep quality [12,13], energy levels [14], mental and psychosocial functioning [15] and quality of life [7,9].

Early diagnosis and timely access to NIV influences an individual’s quality of life and quality of experience using the equipment itself [16,17]. Our previous research has shown that large regional and ethnic variations in access to NIV exist in New Zealand, irrespective of the underlying diagnosis [18]. The reasons for our findings are unclear. However, barriers or failures within the health system are likely contributing factors to this problem. We hypothesize that these barriers to access to NIV may relate to diagnosis, availability of equipment resources and funding, consistency and quality of care provided to establish therapy and maintenance of quality therapy. Furthermore, timely initiation of therapy early in respiratory failure is difficult both due to the non-specific symptomatology (e.g., asymptomatic, or fatigue and/or headaches) and lack of systematic screening for respiratory failure amongst patients.

To date, limited international research has explored why variations in access to NIV exist. Researchers in the USA have noted the complexity of determining NIV therapeutic needs during the variable period of nocturnal hypoventilation preceding daytime hypercapnia, compounded by a lack of a definitive clinical test [4]. Evidence, predominantly from Scandinavia and the UK, has suggested that funding and regional resource allocation may negatively affect the training and confidence of health professionals and the variety of equipment options health professionals can try when establishing and maintaining NIV therapy [17,19,20,21]. In New Zealand, ethnicity, socioeconomic deprivation, and low levels of education have been shown to independently predict decreased uptake and adherence to continuous positive airway pressure (CPAP) in obstructive sleep apnea [22].

Experiences of access to NIV and quality of care from the patient’s perspective are also limited. An international review synthesizing 32 papers identified issues related to the clinician–patient relationship but was predominantly focused on the lived experience of NIV and only a third of the included papers specifically related to people with NMD [17]. A survey of long-term community NIV users, with various health conditions, from one region in New Zealand, identified benefits, but also a few broadly categorized NIV treatment-related problems [14]. Chang (2010) [14] discussed that people with NMD may need extra follow-up due to complications associated with extended NIV use and the progressive nature of the conditions. Whether NIV ‘service provision was adequate’ was variable within their cohort, a result which may have occurred partially based on clinical conditions. However, only 7 (16%) of their 45-person quantitative survey had neuromuscular conditions [14] and the data from this survey is now 12 years old [14]. In short, research has predominantly explored the ‘lived experience’ of NIV, including the transition from non-invasive to invasive ventilation, rather than the practicalities of access to NIV-related quality care, or featured the experiences of people with other types of respiratory failure, such as obesity-related sleep apnea (OSA) and/or chronic obstructive pulmonary disease (COPD), with or without the additional inclusion of people with NMD. Therefore, there remains a lack of in-depth research into the specific healthcare experiences influencing NIV access and uptake specific to people with NMD.

While some of the barriers to accessing quality NIV healthcare and the burden of long-term NIV will be similar across NIV cohorts, it is possible that some factors experienced by people with NMD are distinct. For example, muscle weakness affecting the interface, mouth closure and seal of the mask, ocular complications from air leaks into eyes that do not close tightly; and arm weakness affecting the ability to independently don and doff the mask are all possibilities of issues unique to this population. Differential ‘quality of NIV care’ arising from these issues may result in inequitable health outcomes for people with NMD [18,20]. Indeed, our research has shown that, despite an increase in the overall prevalence use of NIV in New Zealand, there has been a significant decrease in the prevalence of NIV use among people with NMD between 2011 and 2018 [18]. We hypothesize that this difference is attributable in some part to health system factors. An important means of understanding system factors with respect to NIV access and use is by exploring experiences of patients. Indeed, New Zealand’s new Pae Ora (Healthy Futures) Act 2022 legislation requires the involvement of consumers and whānau voices in service and planning [23].

The aim of this New Zealand-based study was to explore the healthcare experiences of people with NMD in order to understand the impact that healthcare system factors have on NIV access and use.

## 2. Materials and Methods

### 2.1. Research Design

A critical realism ontological paradigm with contextualism epistemology was used to approach the research question [24]. Critical realism as a paradigm is increasingly used in health services research to frame, identify and understand complex phenomena experienced by individuals in interaction with their contexts [25]. It can identify mechanisms and processes of the underlying structures and power dynamics that influence individual experiences [26]. The contextualism epistemology acknowledges the sociocultural contexts of the participants and privileges their sense-making of the healthcare setting.

Reflexive Thematic Analysis (RTA) guided the research process and data interpretation. RTA is a robust methodology which embraces the analytical skills, theoretical assumptions and knowledge of the data set theory and resources of the researchers when developing, analysing, and interpreting themes across the data [24]. RTA is also a systematic method suitable for health systems research that integrates the researchers’ analytical skills, their knowledge of the data collected and understanding of the social and organizational factors that occur in health system contexts. This reflexive method allows the researchers to coherently interpret the diverse experiences of participants, identify the underlying mechanisms and make meaningful recommendations to inform policy and practice.

The Consolidated Criteria for Reporting Qualitative Studies (COREQ) guide [27] was used to ensure all requisite details of the research were presented. This study was approved by the University of Otago Human Ethics Committee (Health Reference number H18/005).

### 2.2. Participants

People were included if they were adults aged 16 years old and older; users of bi-level positive pressure non-invasive ventilation (NIV) for over 1 year and with a diagnosis of an NMD. Participants were excluded if they were using continuous positive airway pressure (CPAP) as CPAP is not generally considered NIV because it provides no inspiratory muscle unloading and tidal ventilation remains completely dependent on the respiratory muscles. Patients with motor neuron disease, myasthenia gravis and multiple sclerosis were also excluded due to the different disease progression of these disorders, [28] but also because the experiences of NIV within these types of NMD are more extensively researched [17,29].

### 2.3. Recruitment and Data Collection

Participants were recruited via advertisements within the 16 public and private centers providing NIV in New Zealand, the Muscular Dystrophy Association New Zealand (the nationally representative disabled persons’ organization supporting members with over 70 forms of neuromuscular condition) and the Neurological Foundation (a non-governmental organization supporting neurological research). No direct solicitation was made by the research team; contacting the researchers was left to the discretion of the individual. No participants refused to participate. All but two participants provided verbal and written consent. Verbal consent only was anticipated and was accepted as a reasonable accommodation outlined within our ethics protocol.

Purposive sampling was used to obtain a sample that include a range of ethnicities, variable duration of NIV use, age and gender. Recruitment ceased when theoretical saturation was determined [24].

Semi-structured interviews were conducted either in the homes of participants or over Zoom video conference calls (Zoom Communications Inc., San Jose, CA, USA). Three caregivers attended interviews at the invitation of the participants with NMD. The caregivers ensured the physical and emotional wellbeing of the participants and contributed to data collection by providing prompts or clarification if a participant had difficulty communicating an experience at the discretion of the participant. Written consent from caregivers was obtained for their data to be included.

The interview schedule was developed iteratively by four of the authors (TI, BJ, MJ, and MP) and discussed with the wider research team. Questions were derived from knowledge of the literature, clinical understanding of the respiratory management of NMD, consultation with the Muscular Dystrophy Association of New Zealand and from the researchers’ own lived experience of disability (TI, BJ, and MP), including NMD. The interview schedule was allowed to expand over time, as interviews exposed additional areas to consider with future participants. 

Semi-structured interviews were conducted by MJ and the main topics covered: Experiences using NIV; The decision to use NIV; The process behind obtaining the equipment and; The participant’s and the caregiver’s role in NIV use and maintenance (Appendix A). Although all topics were covered, topics were not always covered in the same order and participants could choose to spend more time focusing on areas of importance to them. The flexibility of interview duration and other reasonable accommodations [30] were offered, for example, to support participants in the management of their fatigue. The services of interpreters (for example, New Zealand Sign language and other language interpreters) were available, but not required. All interviews were recorded with an Olympus DS-55 digital voice recorder (Olympus Corporation, Tokyo, Japan) and transcribed verbatim by a contracted transcriber. Participants’ names were removed and coded as a number (e.g., P1) or as the caregiver for that participant (e.g., P1-CG).

### 2.4. Analysis

The data in this manuscript represent the results from a primary analysis following the six-phase approach proposed for RTA [24]. RTA is highly iterative and reflexive. Therefore, the research team’s collective experiences contributed to the deductive and semantic interpretation of data occurring within and between phases [24]. The analysis was underpinned by an Equity of Health Care Framework, which considers health service delivery from systems (macro), organization (meso) and health professional (micro) perspective [31].

#### 2.4.1. Reflexivity

The interprofessional research team consisted of a respiratory specialist consultant, a public health physician, a nurse, two physiotherapists and an exercise psychologist. The four male and two female researchers all have clinical experience and more than 60 years of combined qualitative research experience. Reflective statements were written by individual members of the research team prior to the analysis process. These included reflection about their own lived experiences of disability, knowledge of and experience with NIV and NMD and values relating to health service delivery. MJ and MP additionally kept notes to document thinking development throughout the analytical process, for example after listening to the audio files, reading transcripts, early coding and research meeting to discuss the data. These strategies enable recognition of metacognition formed from perspectives, knowledge and assumptions about the data interpretation [24,30,31]. 

#### 2.4.2. Familiarization of Data and Coding (Phases 1 and 2)

Transcripts were imported into NVivo (Version 12.6.0; QSR International (UK) LLC, Daresbury, Cheshire, UK). NVivo was used as an aid to organize data within the analysis process. Each audio of the interviews was repeatedly listened to either independently of or alongside the re-reading of the transcripts [24]. Segments of data within each transcript were coded as being relevant, then theme ideas were subsequently developed between transcripts by MJ [24]. In addition, BJ and MP read all transcripts and independently coded five and six of the eleven transcripts, respectively.

#### 2.4.3. Generating Initial Themes, Reviewing and Refining Themes and Writing (Phase 3 to 6)

MJ generated an initial theme list which was reviewed, firstly, with BJ and MP in conjunction with their initial theme lists before being discussed with the wider research team [24]. Joint interpretation of the data and relationships between initial themes was considered by oral discourse and consideration of draft written themes [24]. Theme development evolved following re-reading of transcripts, consideration of interview notes and early independent interpretation notes, consideration of the health systems equity literature, research team meetings and subsequent development of the written results. As part of this process, theme definitions were developed, and themes were ‘named’ [24] and mapped onto the Health Equity Framework [31]. Once the wider team was satisfied with the level of data interpretation, participants were sent an overview of the refined themes and their implications. Five participants responded to this opportunity and no further refinement of the names of themes, their definitions or content arose from this process.

### 2.5. Trustworthiness

A variety of techniques were used to enhance the trustworthiness of this research. We have provided transparent methods including the use of reflective statements and data notes. MJ and MP had prolonged engagement to aid iterative interpretation [24,32]. We employed data and investigator triangulation, as we explored our interpretation with the wider research team and with the study participants providing opportunity for refinement of interpretation [24,32,33]. Importantly, the research team includes academic (PhD) health professionals from several different backgrounds and four of the researchers have lived experience of disability, including NMD, thus providing a particular insight into the data interpretation and analysis. We also provided an in-depth description of the participants. These two factors support inference transferability [24].

## 3. Results

Eleven participants were interviewed within the age range of 20–64 years old (Table 1). Four participants were in their twenties, with the remaining participants equally distributed between the third and sixth decade in age. Six were female. Nine participants identified as New Zealand European, two as Māori and one as Chinese. Participants could self-identify with more than one ethnic group. Three had metabolic disorders and eight various muscular dystrophies. Specific conditions included: Emery-Dreifuss muscular dystrophy, Facioscapulohumeral muscular dystrophy, Limb-Girdle muscular dystrophy, Pompe disease, Centronuclear myopathy, Myotonic dystrophy, Congenital myopathy, Mucopolysacharroidosis type-IVA, and Duchenne muscular dystrophy. All lived in their own home environment (i.e., none lived in residential care). Participants had used NIV for between 2 and 27 years. Three participants used NIV for nocturnal use only, seven for nocturnal and intermittent day use, and one participant used NIV for more than 15 h per day (NIV dependent). Interviews lasted on average 60 min. 

The conceptual themes are presented chronologically to reflect the patient journey and are as follows: Uptake and informed consent for NIV therapy; practicalities of NIV use; and patient–clinician relationships. Within each of these themes, sub-themes, where relevant, are also identified. The themes are summarized in Table 2. In keeping with our method, the themes are thematically mapped to the Health Equity Framework [31].

### 3.1. Uptake of NIV

#### 3.1.1. Informed Consent: “*There Was No Deciding… We Just Had to Accept*”

Individual circumstances played a part in participants’ initial access to and uptake of NIV. Age at the time of NIV prescription also influenced uptake. Four participants were prescribed NIV as adults (Participants 3, 4, 7, 11) and described the choice as their own, even if they did not know of other options. They suggested that NIV was just one of many therapies required:

*“When you leave hospital you’re laden with all the prescriptions you need and... that’s [NIV] just one of them”* (P2).

For one adult participant, acceptance of the equipment was ‘primed’ due to the death of her brother. She discussed that her brother, who had a similar neuromuscular condition as herself, had experienced a delayed clinical decision regarding the need for therapeutic NIV and suggested that this was a factor which had contributed to his early death. The participant stated that this incident “*took away all of that initial ambivalence*” (P4) regarding NIV uptake and described it as a “*relief*” when NIV was offered to her.

Participants that began NIV treatment during childhood reported their parents’ (typically as their primary caregivers) involvement in the decision, with them having limited awareness of the breadth or depth of any discussion occurring:

*“When I first got the BiPAP I didn’t really have an idea what was happening because I was so young and I turned up and it was there”* (P9).

Regardless, most participants reported a lack of discussion on the initiation of NIV therapy with several making comments, such as “*There was no deciding… we just had to accept it*” (P5) and that in this decision they were not considered the experts:

*“I did not decide…that has been all left up to the experts.... I mean they didn’t consult you at all”* (P7).

Discussion about other potential therapy options was also limited, “*It wasn’t discussed whether that was the only option, they said ‘we’ve got this for you’*” (P4). This was especially true if NIV was initiated as part of acute care. For example, one participant was in a coma due to respiratory failure when he first began NIV treatment, and so NIV was initiated as a life-preserving treatment:

*“I ended up in a coma for six weeks and got the right treatment [including NIV] and survived”* (P8).

#### 3.1.2. Establishing a Clinical Need for NIV 

Initial assessments required for determining the need for therapeutic NIV were considered burdensome and a ‘battle’, particularly by participants living in the community. This aspect of care was not so troublesome for participants who were first given NIV in an acute setting. One described traveling for over two hours each way to these assessments, which she described as “*not suitable*” (P7), as it impacted her quality of life and placed a burden on family. Caregivers described the battle with clinicians to establish the need for NIV, a process that took over a year:

*“It was a battle. No one would listen to me because they are saying “His sleep study was fine”* (P10-CG).

Undertaking a sleep study, including invasive assessment procedures, was arduous and painful. Participant 1 and her caregiver described the experiences of arterial blood gas (ABG) testing:

*“CO_2_, they normally check that using ABG’s. she was about a week or so in hospital going through a pile of different tests which is very traumatic leading to not wanting painful treatments in the future…She got to a point where she wasn’t willing to have any more ABG’s because they were making such a meal of trying to get the blood, the inexperienced people taking the bloods. So, she refused in the end, and so I said… well I had a look at Dr Google and realized that there were other ways of doing it, and trying to you know, get them to use some other method”* (P1-CG).

The participant noted that the arterial blood gas should occur immediately upon the patient waking after an overnight hospital or sleep center stay (to resemble blood gas levels found during sleep). However, she stated that her blood gas draw was often delayed due to staff shortages or equipment failure, resulting in the test occurring 30–60 min after waking. She believed this created a falsely more favorable result; she was consequently relieved when therapeutic need was finally established:

*“Our introduction to the BiPAP machine was in the hospital, but not as well supported as it might have been as the NIV nurse specialist goes off at four or five [pm]... there’s no non-invasive specialist on overnight....but it was a relief to get a diagnosis that said this is one of the causes of your problems.... it will help to clear the CO_2_... so we were both pleased about that, and persistent about getting the machine”* (P1-CG).

#### 3.1.3. Establishing an Effective Therapeutic NIV Setting

Few participants discussed specific experiences around establishing an appropriate therapeutic setting but those that did either said it was “*awesome*” (P4) or that it took “*It felt powerful, it took a while to get the settings right*” (P8). For, Participant 4, the therapeutic setting was easily established:

*“I went to bed, and I woke up in exactly the same position 12 h later, did not move or wake up and that was my first experience at home; pretty straight forward, really was awesome getting that first machine”* (P4).

For those that required setting adjustments, this seemed to occur either after a period of continuous NIV, such as following acute respiratory infections, or when the balance between required inspiratory pressure needed to be balanced against discomfort. These scenarios were discussed in detail by Participant 5 after being in an intensive care unit and by Participant 8

*“When I went in, I ended up on life support, then they weaned me off that...so, the first week I had to have it [NIV] on all day…and for sleeping with it at night…Then they started to ween me off and you know it was just trial and error…I would go off in the morning on in the afternoon, or on in the morning, off in the afternoon, off for a bit and then on again at night…that was for about a week or so…then from there I went home and I did the same thing until it was just at night”* (P5).

*“The first two nights I had it on that pressure, I thought ‘oh my goodness, my chest is gonna explode’ and it was just so tight. But now it’s just very relaxed and if I need to take a deep breath I can, whereas before the machine was cutting me fractionally short”* (P8).

Adjustment of NIV pressure was also required due to other side effects such as bloating and flatulence, and this, too, could take some time to resolve:

*“When I first got it put on, they did reduce it [the pressure] ‘cause there was quite a bit of bloating happening… the only real downside of the machine is the air that it pushes into your stomach”* (P2).

Remote monitoring of NIV clinical effectiveness was discussed by Participant 3 only, but this technology is slowly being made more widely available across New Zealand. 

### 3.2. Practicalities of NIV

#### 3.2.1. Equipment Set-Up and Care Instructions

The actual physical process of obtaining the equipment and initial set-up, once NIV was deemed clinically relevant, was considered straightforward. Most participants indicated that the ‘technical’ equipment was user-friendly and intuitive:

*“If you’re used to using a cell phone, you can use one of those [NIV machines]”* (P2).

However, maintaining the NIV, such as instructions on cleaning were ad hoc. One caregiver said she was given two training sessions. Participant 2 received comprehensive equipment demonstrations, Participant 7 was simply provided with an instruction booklet, while another participant said that she was not instructed in terms of setting up or maintaining equipment hygiene at all:

*“[I] didn’t have care instructions… I can’t really remember, didn’t have anything written down”* (P3).

Only when this participant visited another clinic in a different city was she instructed on how to clean the equipment properly:

*“The nurses… they told me and wrote out a list of what needs to happen”* (P3).

#### 3.2.2. Getting the Right Machine and Mask

It was evident that getting the ‘right’ machine and mask affected not only uptake but also therapeutic effectiveness. Some participants discussed their experience of NIV machines without dehumidifiers, which caused a build-up of condensation ‘rain’ on the face. Due to upper limb weakness, some participants were unable to independently remove the mask. They feared the risk of infection due to water pooling in their lungs. Participants demonstrated innovative problem-solving skills to mitigate the issue. However, the ultimate solution was an NIV model with a heated air hose:

*“There is the issues that are fundamental to the equipment, like rain... Condensation! It builds up in the tube and makes a noise and at that point I call Mum and she can lift the tube and the water goes back into the humidifier. If I am asleep, it does eventually cause water into my lungs”* (P4).

*“We had considerable trouble with moisture collecting in the tube. We insulated it. We heated the room. You know we did everything we could think of, but we still had this issue with moisture collecting in the hose... condensation and that would mean that [Name] would get moisture in the mask which would rain on her face...and into her lungs. We eventually got an upgrade which had a heated air hose which meant that [Name] stopped being rained on”* (P1-CG).

However, all participants explained the importance of establishing a good mask interface. They complained of the frustration and pain from pressure sores from ill-fitting masks and other concerns, such as the shifting of teeth from the mask pressure were. The ideal mask was one “*that floats on the person’s face*” (P1-CG), but this “*is not what happens at all*”. Participants discussed their ‘trouble with masks’, the pressure marks that were left and how tight the straps needed to be to ensure a suitable interface (i.e., no leaks and comfortable fit while ensuring therapeutic effectiveness):

*“Nothing but trouble with masks. People say I have marks around my nose from [healed] pressure sores caused by the first mask. Now, lots of pressure marks and sore, but I’m picking because I haven’t got the facial muscles, it tends to leak quite a lot of air; [there] is one [mask] I want to try and get, one with a memory foam cushion but I’ll have to fund it myself”* (P2).

Participants suggested that skin breakdown arose especially “*around the cheeks when the pressure gets up and the face muscles are flaccid*” (P4), yet there appeared to be a one size fits all attitude from health professionals, with comments such as “*this mask fits 95% of our patients*” (P1). Participants explained that no other option was made available unless participants either strongly advocated for a new mask or developed pressure sores (which were not only painful but also reduced the seal and thereby minimizing therapeutic pressure):

*“The first mask I had, one over my nose and I ended up with skin coming off and bruising and bleeding...I asked for a new mask, and they didn’t give it to me... the support worker who just took pictures of my nose ended up sending it through to say ‘I can’t live with this, it’s painful’... and then all of a sudden, ‘Yep. We’ll find you another mask’”* (P3).

Whether additional or replacement masks were funded, or whether only one type of mask was funded, differed by geographical location. This was problematic, as not all participants could afford to pay for a mask or for a customized fit that met the participant’s therapeutic needs without creating further health issues:

*“The mask you have to pay for yourself. When I was doing it through [city] the gear was supplied. Going through the Respiratory Clinic here in [different city], you have to pay for your own masks and bits and pieces…but if you get into a situation that your life capacity’s reduced, then your energy levels decrease and to quantify it, your earning capacity as well, so when you have to go out and fork out $300 for a new mask, it can be a challenge...But it’s not something you can go without”* (P4).

Long-term use of a mask was also linked by participants to changes in the shape of their jawline caused by decreased muscle tone around this area. These long-term effects impacted their ability to eat and sometimes required corrective surgery, which was also costly:

*“The risk of a mask that is meant to sit between the knob of your chin and the bottom of your lip, knocking your back teeth out of place”* (P1).

Another aspect related to NIV acceptance was becoming accustomed to the aesthetics of the mask. Participant 5 was a teenager when she started on NIV and the look of the mask took some time to adjust to:

*“It was a full face [mask] you know. It was friggin’ ugly... it was terrible. I cried, and I said to my Dad, ‘No, I don’t want to wear it Dad!” …I used to not like looking at myself in the mirror, it used to make me feel funny… but today it is part of my everyday life, just something I have learnt to live with. I can certainly say, I can’t live without it um, so it just is”* (P5).

#### 3.2.3. Maintenance, Servicing and Upgrading of Equipment

Participants were somewhat uncertain about the day-to-day maintenance of their NIV. They knew their machine should be cleaned regularly but were not consistent in their descriptions of what or how this should be done. Most did report cleaning it or said that others helped to clean it for them:

*“The first one had an external humidifier tank that sat, fitted across an element…Then they changed it, and they had this big piece on the front, well, I couldn’t get that on and off it was just too difficult, [Caregiver] had to do that for me. …This machine I have got now I don’t really need any support because it is so easy to use, replenish the tank and things is so easy that I don’t actually need any support around that”* (P7).

Maintenance of the filter systems was another aspect participants were expected to manage, but the number of filters sent by the hospital intimated that these should need replacing only intermittently.

*“There is no other maintenance really other than the hose, and everything needs to be cleaned. My partner actually does that, I do it occasionally…the mask they say to wash everyday but I don’t, I just wipe it with an antibacterial which isn’t provided. The filter is always dirty, not dirty but needs replacing and they never suggest replacing them unless we ask for the filters, and then they send you like two filters”* (P8).

All the participants were aware that their NIV machine required routine servicing, including electrical testing, calibration, cleaning and filter changes. Participants were given the impression that it was their responsibility to ensure their NIV machine was serviced rather than this being routinely initiated by the service provider:

*“It is up to us as the patient to chase up when they service it… they have actually warned us at the hospital in [city], they told me ‘Don’t let it go over the overdue date…we are liable, it is not good for us’”* (P8).

That said, other participants found their hospital very responsive to unexpected servicing requests:

*“Recently the on/off button was getting a bit dodgy, you had to push it a few times, but I was down at the hospital, I popped in there and they said, ‘ok we’ll have a look at that’, had it back to me within the space of an hour”* (P4).

Servicing provided participants with an opportunity to experience different NIV machines. None of the other participants expected to have the latest NIV model, and only one participant had a new NIV model, but they knew from their support groups (internationally and nationally) what machine options were available. For example, NIV Participant 2 currently has is smaller and more portable machine that matches her lifestyle:

*“I got a new machine which is smaller and better, and it came with a battery pack, so it didn’t have to be plugged into the wall.... So, I have been able to use it when I go on long road trips, I am able to use it for a bit in the car. It comes in a nice bag and it all just packs away in there and you can take it on the plane, and it doesn’t count towards your luggage allowance”* (P2).

However, more often, participants described the “*reality is [that] they are usually older than the ones they have given you before*” (P8). This meant that participants sometimes received a machine they were unsure how to use or one that did not have the expected safety features to ensure participants and caregivers had a good night’s sleep. In one case, a participant described a community-based replacement service. However, the therapeutic settings on the device had not been programmed correctly:

*“More often [company] would turn up on the doorstep and say, ‘Your machine’s due for service, you take this one and we’ll take yours’. The first time that happened I was agreeing. I trusted the system… but the setting on the machine weren’t right and it didn’t have a humidifier nor an alarm for if the power went off…and I need to know that the powers gone off if I am asleep so I can physically plug the battery in because its putting [P1] at risk so, once we realized that, then the next time it was due for a service I made the guy wait to check that the machine he was leaving was up to scratch before I let him go”* (P1-CG).

Servicing was considered especially important by those with outdated equipment: *“We are still out of date with what we should have… The [hospitals] don’t really have the funding…and they tell me that themselves”* (P8). Outdated machines were also perceived to be more likely to fail. When equipment failure did occur, there appeared to be no easy method for quick replacement and could necessitate a hospital admission to access NIV. This increased the burden on participants, as only tertiary respiratory services based in the larger centers had the expertise to provide acute NIV as an inpatient and, secondarily, to replace the NIV machine: *“I rang the hospital here and they don’t even know what the machine was”* (P8). This led to a four-hour drive in the middle of the night from Participant 8’s rural location to a larger treatment center.

#### 3.2.4. Resource Allocation

Equipment provision was variable across the service providers participants were assigned to. Ensuring power supply in the event of a power outage was a consistent anxiety for all participants, as the inability to independently remove the mask in such an event could lead to suffocation. Some participants were provided with a backup battery by their respective hospitals, while others were required to self-fund such equipment. This financial burden was often not met:

*“I don’t have any life support if the power goes off, nothing, there is no back up no battery, that is up to you to buy. It [lack of battery back-up] is a bit of a concern because it is $700 to buy a back-up device”* (P8).

*“If there’s a power cut. She’s not aware at all. Nothing in this house will beep at her and tell her that something’s wrong”* (P6-CG).

Consequently, participants described resourceful and innovative solutions to ensure ongoing power in the event they did not have a battery backup, “*I’ve got a generator but it’s an old one that needs petrol*” (P3). The necessity of an uninterruptible power supply and reserve battery was evident from the dire implications participants envisioned of suffocating from a lack of air due to mask occlusion (i.e., the inability to independently remove the mask) in the event of a power failure whilst asleep:

*“I needed to get a battery backup one because I thought ‘I am not going through this near death [experience] again!’”* (P6).

The reality of the participants’ vulnerability was reinforced via experiences when a backup power supply was not readily available. For example, when participants required acute care, resulting in the magnification of an already stressful event:

*“We didn’t have a backup battery… we ambulanced her in and they didn’t have a back-up AC power…we fortunately had a connector to the cigarette lighter…otherwise she couldn’t breathe”* (P6-CG).

For the participant who was dependent on NIV, the lack of a battery was particularly pertinent. Consequently, Participant 10’s parents developed a system of manual breathing that involved them repeatedly lifting his arm up and down to approximate breathing, which, while effective, was neither a dignified nor long-term solution.

### 3.3. Patient-Clinician Relationships

Rapport between participants, health professionals and caregivers was considered important for ensuring quality NIV support. This was Partly due to the duration of the relationship, with familiarity enabling more personalized care. “*They know me… and so they have got a better set up*” (P9). Similarly, Participant 6’s caregiver also stated that:

*“That whole relationship, for 18 years we have been going up there [to the clinic] … that was really important for continuity [of care provision]”* (P6-CG).

However, this same caregiver, who was also a trained nurse, believed that knowing how to *“manage conversations”* with clinical staff was important for receiving good care. The ability to ‘manage’ the relationship with health professionals was considered particularly important, as participants requested new or additional support, or were concerned about an aspect of NIV, as they did not want to compromise their quality of care. Participants perceived that relationship management was awkward, time and energy consuming and paternalistic, especially when participants perceived that some health professionals were indifferent about their machine and equipment concerns and perhaps not cognizant of the underlying anxiety and physical discomfort participants were experiencing. 

When Participant 2 raised concerns regarding his lack of reserve power supply, the response was dismissive, “*They didn’t seem to be too concerned*.” Participant 1 described the painful experience of an ill-fitting mask which was also dismissed:

*“When we said to her ‘[Participant 1’s] got this pressure sore’ she said ‘Oh, that [is] nothing. I’ve got a patient who has got it down to the bone”* (P1-CG).

There also seemed to be little understanding or empathy that once a pressure sore arose, the management of the sore while maintaining prescribed NIV became more complex not only for the participant, but also for caregivers, as managing the wounds required time:

*“He has actually got a pressure sore on his nose, so I will have to address that daily now”* (P10-CG).

Some health professionals forgot that, as younger participants aged, their capability to make their own decisions changed. Participant 6 describes her mask concerns being disregarded even though she was old enough to decide. “*They [medical staff] tried to get me to wear the full mask… and they are looking at mum to change my mind.*” This was supported by the participant’s mother, who stated:

*“One thing I thought that they [medical staff] forgot is that the children with chronic illnesses grow up… I think they keep them little and don’t give them room to say no, or to negotiate”* (P6-CG).

Inevitably, participants found that these sorts of interactions and ongoing dialogues wearing. Consequently, having a strong advocate was essential to ensure equitable and person-centered care:

*“She is my full-time caregiver and constant companion. She is with me at all my medical appointments, and she does the nasty phone calls when they need to be made.... I find it very difficult to advocate for myself because I get so upset with it all (and short of breath) … I can do without that. So, it is a source of anxiety”* (P1).

## 4. Discussion

The aim of this research was to explore people with NMDs experience of NIV. This study specifically looked at the practicalities of establishing the clinical need and uptake of NIV and service provision. Participants described a range of similarities and differences in their experiences of Uptake and informed consent for NIV therapy, practicalities of NIV use and Patient-clinician relationships, demonstrating systemic issues occurring across levels of the health and disability system.

### 4.1. System (Macro) Level Issues

System (macro) level issues were evident with the use of different clinical criteria to determine NIV therapeutic need, and discrepancies in equipment provision were evident between different areas of New Zealand. Given that NMD conditions are progressive, and that therapeutic dose adjustments and titration are important, only one of the participants in this study had an NIV machine with remote wireless monitoring to determine clinical effectiveness. This lack of remote monitoring necessitated semi-regular burdensome and invasive studies to determine new dosage parameters. 

Most NIV machines did not have internal battery backup nor an alarm system for power outages while others did. This meant participants in some areas of New Zealand were privately paying for costly battery backup as well as other consumables, such as masks, while participants in other areas of New Zealand were not. Furthermore, some families were being burdened by anxiety and sleep deprivation due to fear of a night-time power outage while asleep and not being able to respond to either remove the mask or set up alternative power options, either independently or on behalf of the person they cared for. 

There were also discrepancies raised between participants in the training they were given and the opportunities for further education on how to maintain the NIV equipment. Finally, the routine use of a written safety plan for power interruption or machine breakage was not evident and this posed a significant risk to the lives of people with NMD in New Zealand. In addition, these sorts of safety concerns are unique to people with NMD, as this population may not have the strength or ability to independently remove the mask or call for help If such a situation arose. 

Few studies have reported systems-level inequities before in New Zealand. A previous survey of a community of NIV users from a single center identified the need to improve service provision, which is greatly needed in New Zealand [14]. Our more recent New Zealand study also demonstrated system-level disparities with NIV service provision between geographical areas but also types of population in New Zealand [18]. However, none of these New Zealand studies specifically aimed to understand how these system-level NIV issues were experienced from the NMD patient perspective. 

International research has identified the difficulties in establishing NIV clinical need and therapeutic dosage [4]. While these issues and others, such as variable education to support initiation, establishment of home NIV, cleaning and maintenance have been reported previously, these papers have not explicitly identified these experiences as examples of system-level issues for people with NMD [4,17,19,34,35]. Ensuring that health professionals provide a minimum standard of education to patients and caregivers is associated with greater tolerance of NIV, decreased burden on caregivers and enhanced therapeutic adherence [17,22,34]. Establishing nationwide expectations and standardized availability of equipment, clinical and funding guidelines for NIV provision for people with NMD, along with training and service standards would be a key opportunity to address these system-level issues.

### 4.2. Organizational (Meso) Level Issues

We clearly identified issues occurring at the organizational (meso) level, particularly the effects of equipment issues (i.e., providing the effective masks) and staff shortages. It appeared that only certain masks were funded, and that there was limited time and attention given by staff to the complexity of mask fitting for people with NMDs. The presumption that a single mask type, aimed at ‘the majority’ of the population would be fit-for-purpose was discussed by all participants. However, we argue that this is an unreasonable assumption given the progressive condition-related muscle changes which can occur with NMD. Furthermore, the experiences described demonstrate the antithesis of person-centered care. It is unclear whether staffing shortages resulting in time constraints and organizational financial constraints (or both) were barriers to providing a comfortable mask that maintained the therapeutic effectiveness of NIV intervention. 

Previous international research has demonstrated similar mask-related issues and described the mask as being frightening, unpleasant, difficult to manipulate and a source of pressure sores [17,36]. Limited research, however, has explored issues with equipment, including masks, by condition type. Fung (2015) [36] did find that people with sensory impairment found the use of the NIV machine, mask donning and doffing and mask-wearing more challenging. Recently, a systematic review of 12 studies identified that the cumulative duration of mask use and changes to the face from oedema were risk factors for pressure injuries [37]. These studies provide supporting evidence to our current study that NIV equipment and mask usability need to be analyzed by population sub-groups, as certain NIV populations are likely to have greater cumulative mask wear time and specific condition-related issues compared to other NIV user populations (e.g., COPD or OSA). 

In New Zealand, mask fitting and setting up of the NIV machine may be undertaken by a variety of professions, but predominantly respiratory physiologists who may find that some skills needed are outside their professional scope of practice. Interprofessional training, which specifically covers the use of NIV by people with NMD, may be one means of improving consistent delivery of a quality health service. It may also help to mitigate the identified staff shortages which negatively affected the care received by the participants with NMD. Indeed, one RCT has demonstrated that health professionals can achieve a mask fit with low mask pressure and minimal air leak if given the education and practical training to achieve this competency [19]. Sleep physiologists have core professional competencies related to mask fitting, equipment set-up and monitoring that should be included in work force development. We also strongly recommend that funding of equipment consumables, such as masks and tubes, should shift from the organization (meso) to the system (macro) level, thereby reducing the need for health professionals to have to negotiate good practice. 

### 4.3. Health Professional (Micro) Level Issues

We also identified examples of issues occurring at the health professional (micro) level. Research has shown that patients’ perceptions of the quality of healthcare are dependent on the ability of the support institution and the helpers to provide individualized care [34]. Participants predominantly described a lack of ‘informed’ consent with an emphasis on the word ‘informed’, as participants seemed to neither know of the alternatives to NIV nor the benefits and risks of NIV. The age of NIV initiation did influence this, with older participants appearing to have more control over the decision, albeit still with limited ‘informing’. Consent was also influenced by circumstances leading up to the need for NIV, with acute respiratory admissions appearing to provide less opportunity for consent. Dreyer (2010) [38] and colleagues argued that health professionals should attempt to support patients’ autonomy wherever possible, but also be prepared to practice soft paternalism when decisions are difficult, sometimes even by making the decisions for patients [38]. They further suggest that the question of whether patients want home ventilation is rhetorical, because ultimately people want to live—which therefore means they require ventilation [38]. These sentiments were echoed by the participants in this study and are reported elsewhere [29]. People requiring NMD may find it a relief when health professionals make these types of difficult decisions for them [17]. However, the utility of such paternalism is debated. For example, it has been argued that paternalism undermines self-determination, self-efficacy and the intent of NIV therapy [6]. An important aspect is each persons’ health literacy—understanding what their decisions entail and their consequences. Without a full understanding of the implications of accepting NIV, it is arguable as to whether true informed consent has been given. Assessing individual health literacy, and subsequently ensuring that information is conveyed at a level appropriate to their health literacy, is a process that should be undertaken by health professionals in charge of presenting NIV as a treatment option. It is by these means that “Respect for inherent dignity, individual autonomy including the freedom to make one’s own choices, and independence of persons”, which is a key principle of Article 3 of the United Nations Convention on the Rights of Persons with Disabilities (UNCRPD), can be upheld [30].

Familiarity and rapport were identified as being important to enable access to resources and for ensuring the quality of care. This seemed to be easier for participants who had been diagnosed with NMD and started NIV at a younger age. Arguably, people with NMD who have not yet had time to develop these relationships should not receive a lesser standard of care. It was also evident that, even with established relationships, participants with NMD were fatigued with the persistent need to advocate for their rights. Some participants in this study said they had caregivers who were able to take on this role on their behalf, while others did not. The review by Ngandu et al. [17] also identified health professional care deficiencies, with some participants experiencing neglect and not being involved in their treatment, which intensified suffering [39]. Our study highlights that these experiences should not only be considered as relationship issues, but should also be considered as another factor leading to differential health outcomes. Previous research has shown that poor health professional relationships can negate the effectiveness of the therapeutic intervention [40]. 

### 4.4. Strengths and Limitations

The study explored participants’ experiences from various geographical areas of New Zealand, which is important when gaining insights into system-level issues. The analysis was underpinned by a well-established Health Equity Framework and our study is novel in its being the first study in Australasia to use this multilevel approach to explore the adequacy of NIV healthcare provision for people with NMD. 

We have provided a strong methodological rationale for presenting these data via Reflexive Thematic Analysis and outlined the steps undertaken to ensure methodological rigor [32,33]. However, this research does have some limitations. 

Our results are generated from the interviews of the participants included in this research and our interpretation of the data. Experiences of people with NMD who have not yet managed to access NIV are not included in this study, so there are potentially additional factors affecting access amongst this group which we have not captured. Therefore, the impact of the barriers that have been identified is conservative.

Despite using purposive sampling to target ethnically diverse participants, the sample obtained was predominantly NZ European. Our recent research has demonstrated, however, that there is a low prevalence of Māori (9.7%), Pacific (2.4%) and Asian (6.5%) peoples among patients with NMD receiving NIV, with lower age-standardized rates at a population level [18]. We also know that different ethnicities experience health delivery in different ways, including in New Zealand [35,41]. Future research should specifically explore the experiences of these groups with culturally appropriate methodology. 

There was agreement between participant experiences, which provides credible narratives on their health experiences in New Zealand, with our data triangulation revealing no divergent perspectives. Our results may apply to other countries’ health systems, but further research to establish the consistency of factors internationally would be required. Nevertheless, we contend that using a health systems framework, such as the Equity of Health Care Framework [31], is a useful and innovative way of considering experiences of health delivery for people with NMD, irrespective of country. 

### 4.5. Implications and Recommendations

The current research brings to attention areas in the health system affecting uptake, provision and support for people using home-based NIV in New Zealand. The World Health Organization has outlined eight key characteristics which should be present in the network of service delivery in any well-functioning health system. These include comprehensiveness, accessibility, coverage, continuity, quality care, person-centeredness, coordination, accountability and efficiency [42]. We found negative experiences in all these areas. New Zealand has just introduced a significant new legislative framework (Pae Ora [Healthy Futures] Act 2022), which unites the previous system of 20 District Health Boards under one provider (Te Whatu Ora/Health New Zealand) along with a newly formed Te Aka Whai Ora/Māori Health Authority. Our research acts as a call to action for policymakers in this therapeutic area. We recommend that Health New Zealand and PHARMAC (The Pharmaceutical Management Agency, which holds accountability for medical devices urgently develop national service specifications with clear standards and dedicated funding for patients with NMD to address the NIV systemic variations and inequities in healthcare we have identified. We also suggest that the New Zealand Ministry of Health and Health Quality and Safety Commission proactively investigates and monitors the variations we have identified. Our research highlighted some of the negative experiences of NIV services experienced by people with NMD in New Zealand. Many of these experiences, especially at system-level, will likely be common to all patient groups, such as the lack of service standards, workforce shortages and equipment availability. However, we have identified several issues which are likely specific to various NMDs relating to the interface—mask fitting with face shape/muscle atrophy and pressure-related complications, as well as the logistics of caregiver training and support. Therefore, research exploring barriers and facilitators to NIV uptake should separately consider the experiences of people with different conditions—including NMD. Furthermore, the age at the time of NIV initiation affected experiences, suggesting that more research including people with diverse NMD, in addition to motor neuron diseases, is warranted. Finally, this research suggestss that mainstream services need to be responsive to disabled people. Therefore, we recommend the development of interprofessional specific disability-related services, as per Articles 25 and 26 of the UNCRPD [30]. As the prevalence of NIV use among people with NMD has risen over the last ten years and is now 4.8 per 100,000 in New Zealand [18], acting on these recommendations should occur immediately.

## 5. Conclusions

This study explored the experiences of people with NMD, establishing the therapeutic need for NIV and the practicalities of NIV service provision in New Zealand. We identified, with the Equity of Health Care Framework, issues at the system, organization and health professional levels, indicating that current NIV service provision would not meet the characteristics of a well-functioning network of health service provision. We recommend the development of national service specifications with clear standards and dedicated funding for patients with NMD and call for the Ministry of Health to proactively investigate and monitor the variations in service delivery identified. The specific areas of concern for patients with NMD suggest the need for NMD-related NIV research and service provision responsive to the distinct needs of this population.

## Figures and Tables

**Table 1:** Demographic details.

Participant	Age (Years)	Duration of NIV Use (Years)	Caregiver Present
1	37	6	Yes
2	52	5	No
3	41	5	No
4	52	20	No
5	64	27	No
6	20	2	Yes
7	64	5	No
8	28	17	No
9	23	5	No
10	29	12	Yes
11	32	5	No

**Table 2:** Themes and Sub-themes mapped to the Health Equity Framework.

Conceptual Themes and Sub-Themes		Health Equity Framework Level
**Uptake of NIV therapy**		Health professional (micro)
	Informed consent	System (macro)
	Establishing a clinical need for NIV	System (macro)
	Establishing an effective therapeutic NIV setting	
**Practicalities of NIV**		
	Equipment set-up and care instructions	Organizational (meso)
	Getting the right machine and mask	Organizational (meso)
	Maintenance, servicing and upgrading of equipment	Organizational (meso)
	Resource allocation	Organizational (meso)
**Patient-clinician relationships**		Health professional (micro)

## Data Availability

The data presented in this study may be available upon request from the corresponding author.

## Appendix A

**Table A1:** Semi-structured interview guide.

Question Examples	Probe
**Section 1: Rapport-building and general experiences using NIV**	
How would you describe your experiences with using NIV?	Please describe any positive or negative aspects.
How does using the equipment affect your everyday life?	For example, physical changes, changes in sleep, or personality.
Can you describe any changes you have experienced since using the NIV equipment?	What thoughts or feelings did you have? Apprehension, relief, fears? Did these change from prior to the first use to after?
Can you tell me about your first experiences of using the machine?	
Tell me about the training you received for using the machine.	Were you confident as a result of that training?
Can you tell me about your experiences with support from the equipment providers or local health board?	For example, a specific time you were supported, and how you felt about this support.
Can you tell me about a time when the equipment has gone wrong, and your experiences in getting it fixed?	How did that make you feel?
**Section 2: The decision to use NIV and the process behind obtaining the equipment**	
Can you describe to me the time that you decided to get NIV?	Who was involved in the decision? How did the process make you feel?
Can you describe the symptoms you were experiencing at the time that you were thinking about getting NIV?	
How has your NIV use changed over time?	For example, early prescription compared to current use.
Can you tell about your experiences of obtaining the NIV machine?	What was the process? Did you make contact with someone, or was it offered to you? Was there a long waiting time?
How did this process make you feel?	For example, listened to, supported, neglected?
**Section 3: Caregivers’ roles in NIV uptake and use**	
What role did your caregiver play in your decision to get NIV?	
What does your caregiver do in terms of maintenance of the machine?	
What other types of support does your caregiver provide?	For example, emotional support, physical support? Any other types?
